# Acceptance and commitment therapy for chronic pain: protocol of a systematic review and individual participant data meta-analysis

**DOI:** 10.1186/s13643-019-1044-2

**Published:** 2019-06-14

**Authors:** Jiaxi Lin, Whitney Scott, Lewis Carpenter, Sam Norton, Matthias Domhardt, Harald Baumeister, Lance M. McCracken

**Affiliations:** 1 Sportpsychology, Institute for Sports and Sport Science, University of Freiburg, Freiburg, Germany; 2 Psychology Department, Institute of Psychiatry, Psychology & Neuroscience, King’s College London, London, UK; 3 Department of Clinical Psychology and Psychotherapy, Institute of Psychology, Ulm University, Ulm, Germany; 4 Department of Psychology, University of Uppsala, Uppsala, Sweden

**Keywords:** Individual participant data meta-analysis, Acceptance and commitment therapy, Chronic pain, Subgroups

## Abstract

**Background:**

Acceptance and commitment therapy (ACT) can be effective in treating chronic pain. Despite evidence supporting the effectiveness of ACT, uncertainties remain regarding which subgroups in the chronic pain population are likely to benefit most and least. This protocol describes the application for two meta-analytic approaches, one at the level of individual participant data and the other at the level of aggregated data, from randomized controlled trials of ACT for chronic pain (ACT-CP-MA).

**Methods:**

We will systematically conduct literature searches in CENTRAL, MEDLINE, EMBASE, PsycINFO, and trial registers. Two reviewers will independently select studies for inclusion and data extraction. ACT-CP-MA will include randomized controlled trials with ACT for chronic pain compared to control conditions for adults (≥ 18 years) with chronic pain (> 3 months). We will invite the authors of all eligible trials to share individual participant data. Outcomes will include standardized measures of pain interference, pain intensity, depression, anxiety, health-related quality of life, participants’ rating of overall improvement, and ACT-related process variables. Using the Cochrane Collaboration’s tool and GRADE, reviewers will independently check for risk of bias, quality of evidence, and strength of recommendations. In the individual participant data meta-analysis, we will use a one-step approach where participants are clustered with studies and data from all studies are modeled simultaneously. For analyses, we will use mixed-effects models. Additionally, we will employ a meta-analysis with aggregate data and compare the results of both meta-analyses.

**Discussion:**

This collaborative meta-analysis of individual participant data from randomized controlled trials of ACT for chronic pain versus control conditions will demonstrate how the known benefits of ACT for chronic pain vary across different subtypes of the chronic pain population. The results of the meta-analyses will be based on a comprehensive search of multiple databases and will help to inform future clinical trials and decision-making on the use of ACT in chronic pain and improve the quality, design, and reporting of future trials in this field.

**Systematic review registration:**

PROSPERO CRD42019120901.

**Electronic supplementary material:**

The online version of this article (10.1186/s13643-019-1044-2) contains supplementary material, which is available to authorized users.

## Background

Chronic pain is defined as pain lasting more than 3 months and can be seen as a disease in its own right [1]. The prevalence rates of chronic pain are in the range of 27% in European countries [2], equivalent to international estimates [3, 4]. This condition is associated with a high disease burden in terms of personal suffering, low quality of life, and high economic costs, and is considered as a major health care problem worldwide [5, 6]. According to the Global Burden of Disease Study of 2017 [7], over the 28-year period from 1990 to 2017, low back pain, headache disorders, and depressive disorders have prevailed as three of the top four leading causes of years lived with disability worldwide.

The biopsychosocial approach is regarded as the gold standard in the treatment of chronic pain [8, 9]. Psychological interventions, such as the acceptance and commitment therapy (ACT), constitute a core component within this approach. ACT aims to increase openness to difficult experiences such as pain, as well as awareness of behavioral options, and to facilitate behavior change processes that are in line with personal life values in the presence of these experiences [10, 11]. Various methods of delivering ACT have been shown to be effective in treating chronic pain: either as individual face-to-face intervention (e.g., [12, 13]), group-delivered face-to-face (e.g., [14–23]), self-help books [24, 25], or Internet-based delivery [26–30]. The British Pain Society recommends ACT in the treatment of chronic pain [31] and although ACT has also been implemented in public care for patients with chronic pain in some countries, considerable barriers to implement psychological interventions have been observed [32].

The effectiveness of ACT for improving pain-related outcomes has been supported in several (non-Cochrane) reviews, with the consistent conclusion that ACT appears to be equally effective as traditional cognitive behavior therapy [33–36]. The latest meta-analysis found small to large effect sizes for measures of functioning, anxiety, and depression (SMD = − 0.45, − 0.57 and − 0.84, respectively) [35]. No treatment effects were observed with regard to the outcomes for quality of life and pain intensity [35]. This evidence-base suggests that the effects of ACT, as with most treatments for chronic pain, need to be improved. One way to do so is to broaden our understanding of which patient characteristics and conditions as well as mode of treatment delivery (e.g., internet-based or face-to-face) are associated with treatment effects and which are not [32, 35, 37, 38]. Hence, examining effect moderators of chronic pain treatment would greatly facilitate patient tailored interventions and constitute a crucial step forward in the management of chronic pain.

A few systematic reviews have already investigated this issue. There is one review focusing on ACT and mindfulness-based approaches by Gilpin and colleagues [39], and reviews on other psychological and non-psychological pain approaches [40–42], and with regard to certain specified aspects, such as fear-avoidance beliefs [43]. In the review by Gilpin and colleagues [39], some evidence was found that higher psychological distress or history of depression is associated with greater improvements in mindfulness-based interventions. Equivalent to an earlier review on behavioral and cognitive-behavioral interventions [42], this review found that relationships between demographic variables and treatment outcome were inconsistent and not significant in most cases [39]. In the review on self-management programs in musculoskeletal pain [41], one study showed that higher levels of depression at baseline predicted poorer physical functioning at 6 months [44]. In patients with low back pain, high fear-avoidance beliefs were associated with more pain and/or disability and lower likelihood to return to work [43]. In general, most of the trials reviewed did not include subgroup analyses and most of the trials with these analyses lacked the power to find reliable treatment effects for specific subgroups. Hence, the evidence-base of treatment moderators is inconsistent. Also, due to the high heterogeneity of the included studies, meta-analyses (MA) of aggregated data (AD, hereinafter referred as AD-MA) were not conducted in the above reviews. Consequently, the current state of scientific knowledge on treatment predictors and moderators is fragmented, inconclusive, and therefore difficult to interpret.

A meta-analysis of individual participant data (IPD, hereinafter referred as IPD-MA) may therefore be a reliable method to overcome high heterogeneity across trials. This not only refers to methodological aspects (e.g., choice of predictor, moderator, and outcome variables, length of follow-up) but also to aspects of the study population, such as gender or a specific pain condition. By using IPD, standardization of analyses and reporting of results across studies, direct derivation of outcomes can be facilitated, independent of how these were reported [45]. IPD also increases the power to detect differential treatment effects between individual participants, allowing for additional examination of who is most likely to respond with a wider range of statistical analyses. Given these reasons and the advantages over AD-MAs, the IPD-MA has been described as the gold standard of systematic reviews [46].

Therefore, the present project aims to conduct an IPD-MA and an AD-MA to systematically review studies on ACT for chronic pain (ACT-CP-MA) in order to provide comprehensive insights into the effects of ACT for chronic pain. In detail, ACT-CP-MA aims

1. To provide an updated AD-MA on the effects of ACT on pain interference and other key outcomes in individuals with chronic pain.
2. To conduct an IPD-MA to evaluate the effects of ACT on pain interference and other key outcomes of individuals with chronic pain.
3. To identify

- individual-related effect modifiers (predictive markers): socio-demographic and pain-related characteristics, ACT-related processes,
- treatment-related effect modifiers: traditional one-to-one face-to-face ACT, group-delivered face-to-face ACT, or internet intervention, number of sessions, and
- study-related effect modifiers (recruitment strategy, quality of assessment, control groups)

factors that moderate treatment effects of ACT in chronic pain in the included randomized controlled trials (RCTs). For these analyses based on IPD, we will derive various factors from ACT theory and research on chronic pain and formulate specific research questions [47–51].

Due to the complexity of this research field, this study protocol describes the general aims of ACT-CP-MA. The specific research questions for the moderator analyses will be presented and reported separately in respective future publications.

## Methods

We will conduct ACT-CP-MA in accordance with the Preferred Reporting Items for Systematic reviews and Meta-Analyses statement (PRISMA [52], see PRISMA-P checklist in Additional file 1) and the extension of PRISMA for IPD-MAs [53]. Studies will be selected according to the PICOS criteria [54] outlined in the following and summarized in Table 1.

**Table 1:** PICOS elements of the study inclusion criteria

Participants	Adult persons (≥ 18 years) with chronic, non-malignant pain (duration > 3 months)	
Intervention	Pain-specific ACT, different modes of delivery	e.g. • Face-to-face individual • Face-to-face group • Self-help book with/without guidance • Self-help internet intervention with/without guidance
Comparator	• Active chronic pain-specific (ACT or non-ACT) treatment • Wait-list • Treatment as usual • Attention control • Psychological placebo • No treatment	
Outcomes	• Pain specific: interference and intensity • Emotional functioning: depression and anxiety • Health-related quality of life • Participants’ rating of overall improvement • ACT specific: pain acceptance, psychological flexibility	
Study design	Randomized controlled trials with ethics approval	

*ACT* acceptance and commitment therapy

### Participants

The population of interest consists of adult persons (≥ 18 years) with chronic (duration > 3 months), non-malignant pain. If a study has included both adolescents and young adults over the age of 18 years, this study will be excluded since it can be assumed that the treatment settings for adults and children/adolescents/young adults can be very heterogenous. Inclusion of primary studies will not be further limited to specific clinical subgroups in order to increase power and the ability to inspect the role of individual and study differences.

### Intervention

In this review, we will exclusively focus on ACT-based interventions. Consistent with a recent review on ACT for chronic pain [35], studies will be included only if the intervention explicitly uses the full ACT model. In accordance with a recent review on predictors and moderators in ACT and mindfulness-based approaches [39], the high heterogeneity in the theoretical base of treatment and therapeutic mechanisms can cause differences in individual responsiveness and may explain inconsistencies between studies. Consequently, we will exclude studies with purely mindfulness-based interventions. All ACT interventions regardless of their mode of delivery will be included in the review (e.g., face-to-face, individual, group, self-help book, internet intervention).

### Comparator(s)

We will include trials if the comparison group received either an active treatment for chronic pain (ACT or non-ACT) or a control condition, i.e., treatment as usual, psychological placebo, attention control condition, waitlist control, or no treatment.

### Outcomes

Our selection of outcomes is based on recommendations from the Initiative on Methods, Measurement, and Pain Assessment in Clinical Trials (IMMPACT; [50, 51, 55]) as well as on theoretically relevant aspects of ACT [39, 56–62].

#### Pain-specific outcomes

IMMPACT recommends the use of the Multidimensional Pain Inventory (MPI; [63]) or interference scale of the Brief Pain Inventory (BPI; [64]) as validated self-report questionnaires for pain interference. In case of multiple outcome measures for pain interference, we will favor the MPI due to the higher number of items (nine in the MPI versus even in the BPI) and given that it is the most frequently used outcome measure across studies. If none of these questionnaires were used in the studies, comparable validated self-report questionnaires, such as the pain disability index (PDI; [65]) will be used (randomly in case of multiple measure). The scores of different questionnaires will be standardized to allow all measures of pain to be modeled together.

The following secondary outcome measures must be assessed by standardized measures:

- Pain intensity (numeric or visual analogue scales)
- Depression (e.g., Patient Health Questionnaire (PHQ-9 [66]) or The Hospital Anxiety Depression Scale (HADS [67]))
- Anxiety (e.g., HADS [67], Generalized Anxiety Disorder-7 scale [68])
- Health-related quality of life (HrQol; e.g., short form 12-item survey (SF-12 [69]), Assessment of Quality of Life (AQoL-8D [70]), or the EuroQol (EQ-5D [71]))
- Participants’ rating of overall improvement (e.g., Patient Global Impression of Change (PGIC [72]))
- ACT-related variables (e.g., psychological flexibility the Acceptance and Action Questionnaire-II (AAQ-II [73]), the Chronic Pain Acceptance Questionnaire (CPAQ [74]), or the Committed Action Questionnaire (CAQ [60]))

### Predictors and moderators of treatment outcome

We differentiate between individual predictors, treatment-related moderators, and study-level moderators for treatment outcome. Individual predictors for treatment outcome are variables assessed at baseline before the start of the treatment. The following factors are defined as potential individual predictors for treatment outcome in this study: socio-demographic factors (age, gender, employment status, level of education, relationship status, comorbid somatic/psychiatric conditions), pain-related factors (pain duration, baseline pain interference, and intensity), and baseline ACT-related factors (e.g., pain acceptance). We identified these factors to be potential predictors of treatment effect from single studies and reviews with inconsistent findings [32, 35, 38–41, 75–81]. Treatment-related factors are the proportion of intended number of sessions attended.

Study-level moderators for treatment outcome include intervention characteristics (i.e., mode of intervention delivery, number of modules/sessions, level of guidance) and study characteristics (i.e., recruitment setting, such as open recruitment through self-referral, or recruitment in primary, secondary or tertiary care). We will also collect information on adverse events and adherence to the treatment as reflected by the percentage of completed modules in each study-specific treatment. We will summarize these data on a descriptive level, given that these aspects are not expected to be reported in all identified studies and as adherence can only be observed in the interventions groups.

### Study design

Randomized controlled trials (RCTs) of any length of follow-up and any setting will be included if reported. We will not apply any exclusion based on publication status, date, or type. We will only use data from studies that received ethics approval and that are published in English.

### Study identification and selection

To minimize publication bias, we will search for published and unpublished trials. For the published trials, we will search for RCTs of ACT for chronic pain in the following databases using medical subject headings (MeSH): (1) Cochrane Central Register of Controlled Trials (CENTRAL), (2) MEDLINE, (3) EMBASE, and (4) PsycINFO. We will use RCT filters for MEDLINE and EMBASE and applied adaptations of these to the other databases. Two reviewers (JL and WS) developed the MEDLINE strategy. The search strategy for the literature search is included in Additional file 2.

Additionally, we will search in the International Standard Randomized Controlled Trial Number register (ISRCTN), WHO International Clinical Trials Registry Platform (ICTRP), ClinicalTrials. Gov, and PROSPERO and examine reference lists of all references of included trials and reviews to identify other potentially relevant studies. Further, we will contact all corresponding authors of included trials and asked about other RCTs, published or unpublished, which might be eligible for the review. These strategies are important in order to find eligible “gray literature” (i.e., unpublished trials, trials, and trials reported as meeting abstracts, book chapters, and letters [82]).

Two reviewers (JL and WS) will independently select relevant studies for inclusion. First, we will examine a list of titles and abstracts. If title and abstract contain sufficient information to determine exclusion, we will exclude the respective article. For all remaining articles, we will retrieve and review the full text independently. In addition, we will review all other potentially relevant articles identified by checking the reference lists or personal communications. If there are discrepancies between reviewers at any stage of the process, we will consult a third reviewer (LM).

### Data collection

We will initially contact the corresponding author of all identified trials to invite them to participate in ACT-CP-MA and to share their raw data (see Additional file 3 for contact letter, a modified version of [83]). This invitation contains a short introduction to ACT-CP-MA, including the aim, inclusion criteria, and outcomes as well as a short description of the ACT-CP-MA procedures. We will offer PIs who have shared their data to become a co-author in the result publications. They are also invited to become active collaborators within the project. If we cannot contact the corresponding author, we will email all co-authors listed. If we fail to receive a response to our initial email invitation, we will send reminders after two and if, necessary, after 4 weeks. If the study investigators are still not responding or unwilling to contribute their study data, we will send a final note inquiring why they are unable to participate. If there is no response after 4 weeks, the trial will be excluded as “unavailable.”

In order to increase the feasibility of the IPD-MA, we will provide regular e-mail updates to keep the collaborative group up to date and involved. On an online collaboration platform (trello.com), we will present all procedures during the acquisition of the data and the analysis and their respective deadlines so that the procedures will be transparent to all collaborators.

We will seek from all relevant trials data for all participants at all assessment times, including those excluded from the investigators’ own analyses. In order to minimize the amount of work for study authors, we will accept databases in all formats. Before transferring the data, we will ask the investigators to anonymize the data and to use a password-protected encryption. Once we have received the original data file, we will archive the original data as backup and transfer the IPD to a converted and combined overall dataset with standardized variables, the ACT-CP-IPD database. For data harmonization, we will apply the procedures described in Buffart et al. [84]. We will discuss any inconsistencies with the original authors and make corrections when necessary.

For the AD-MA, a standardized data extraction form has been developed and will be piloted, based on the template of the Cochrane good practice data extraction form (Version 4, April 2014, see Additional file 4) to extract data from the selected studies.

### Assessment of risk of bias in included studies

Two reviewers (JL and WS) will independently assess risk of bias using the Cochrane Risk of Bias assessment tool, focusing on the evaluation of sequence generation, allocation concealment, blinding (participants, personnel, and outcome assessors), incomplete data, selective outcome reporting, and assessing other biases [85]. Additionally, the researchers (JL and WS) will assess the evidence profiles for each of the identified outcomes based on the GRADE (Grading of Recommendations, Assessment, Development and Evaluation) approach to reviewing evidence [86–88]. In case of disagreement between the reviewers, discussions or consultation of a third reviewer (MD) will take place. For the trials that were conducted by JL or WS, MD will assess the risk of bias instead of JL or WS, respectively. If needed, a statistician (SN or LC) will be consulted regarding judgments relating to statistical analyses.

### Statistical analysis

#### Individual participant data meta-analyses

We will perform analyses according to current expert recommendations for individual participant data meta-analysis [45, 89]. By using mixed-effects models, we will apply the one-stage approach IPD-MA based on the combination of all data in a single meta-analysis [89, 90]. In order to consider clustering effects from study to study, we will apply a two-level hierarchical structure: the participants within each trial as level 1 and the trial as level 2. We will perform all analyses on a modified intention-to-treat (ITT) basis using a statistical interpolation strategy for missing data within the mixed-effects model. For this strategy, we will include all randomized participants with outcome data.

Using mixed-effects models, we will examine treatment effects on the specified outcomes. With regard to potential treatment moderators on the outcomes, we will examine interactions between the intervention and the above-mentioned individual predictors, treatment-related moderators, and study-level moderators. For data management and harmonization, SPSS will be used as most datasets will be available in SPSS format. Analyses will be conducted using R (R Foundation for Statistical Computing, [91]) which offers a wide variety of basic to advanced statistical and graphical techniques.

#### Aggregate data meta-analyses

For those studies where IPD were not provided, a sensitivity analysis exploring the estimated treatment effect with AD will be conducted. To estimate the treatment effect of ACT compared to different control groups, we will calculate the effect sizes (Hedges’ *g*) [92], which will be pooled using a random-effects meta-analysis. We will test for statistical heterogeneity using the Chi2 test (significance level: 0.1) and *I*^2^ statistic on the basis of the Cochrane Handbook [93] recommendations. To examine publication bias, we will visually inspect the funnel plot, by using the trim and fill procedure and Egger’s test of funnel plot asymmetry [94, 95]. A sensitivity analysis will also be conducted excluding studies with high risk of bias.

This AD-MA will exclusively examine treatment effects on the specified outcomes without investigating potential treatment moderators or predictors. This decision is based on the lack of studies investigating these variables in ACT and mindfulness-based treatments for chronic pain as documented in a recent meta-analysis [39]. Further, for variables measured at the individual level, the IPD-MA will have considerably greater power to investigate this research question in comparison to an AD-MA.

The results with regard to the treatment effects of both meta-analyses will be compared: By doing this, we will analyze and discuss potential differences between studies included in the IPD-MA and those not included.

## Discussion

ACT-CP-MA will use a thoroughly defined methodology and provide an updated review on the effect of ACT for chronic pain. It will be the first study that comprehensively examines data with an explorative study approach including important potential predictor and moderators of treatment effects. Therefore, it can overcome predefined hypotheses that were applied in each included primary study and allows for an overview of factors that may be crucial to treatment effects observed in ACT for chronic pain.

The central strength of this study lies in the methodological approach which will use an IPD-MA of randomized controlled studies on ACT for chronic pain based on an extensive search of multiple databases, journals, references, and citations. We will deal with a wide array of outcomes based on theory and experts’ recommendations as well as with individual-based predictors and treatment-related moderators of treatment effects. Using the same systematic procedures, harmonization of variables and analyses on these variables across multiple studies, we will provide a consistent evidence synthesis across all variables. Results of this review will be published in international medical and psychological journals and presented at national and international conference meetings following the PRISMA statement [52, 53].

### Limitations

We will possibly face some difficulties in obtaining original data from all of the identified trials. These difficulties may result in a bias to the IPD-MA [45]. Therefore, we will additionally conduct an AD-MA with all identified studies. This AD will provide a basis for comparisons between the AD-MA and IPD-MA. Furthermore, we will increase the exchange in the field of research on ACT and chronic pain in order to build a collaborative network to facilitate the exchange of data.

## Conclusions

In ACT-CP-MA, we will review RCTs of ACT for chronic pain and bring together relevant evidence. We will discuss the findings with regard to future directions of research on ACT for chronic pain as well as implications for health care services to help people make well-informed decisions. The information on different treatment predictors and moderators may help clinicians match patients most likely to benefit from ACT with this treatment approach in the sense of personalized medicine. This will also allow conclusions on which characteristics of individuals would best benefit from which form of intervention (face to face, internet-based, group-based, individual). Thereby, treatment costs may be better controlled by allocating treatment resources where they are likely to be most effective [42]. Therefore, the results of this review may provide a basis for treatment guidelines for chronic pain patients with respect to the provision of ACT and its type of delivery. The findings of this study will help researchers to refine ACT-based interventions for chronic pain. In the development phase, knowledge of user characteristics that are linked to greater improvements can be used to enhance the efficacy of ACT through greater targeting of treatment toward those characteristics.

The IPD-database of this project is conceptualized as a basis for other MAs to come in the future. As not all research questions might be answerable due to the unavailability of the current evidence, this project is designed as a long-term project in which we will continuously update the IPD-database and subsequently provide more in-depth analyses on the effectiveness of ACT in chronic pain patients. Different researchers within the collaborative network of this project can investigate different specific research questions. For example, more knowledge on treatment processes within ACT and patient characteristics can be an essential step forward to improve treatment effects for chronic pain [96–105]. The collaborative structure of this project will also stimulate scientific exchange between experts in the field which might generate ideas for methods to improve future trials or treatment development.

## Additional files

Additional file 1: ACT-CP-MA PRISMA-P checklist. (DOCX 31 kb)

Additional file 2: ACT-CP-MA bibliographic database searches. (DOCX 20 kb)

Additional file 3: ACT Pain IPD invitation letter. (DOCX 26 kb)

Additional file 4: ACT-CP-MA data extraction form. (DOC 187 kb)

## Abbreviations

ACT: Acceptance and commitment therapy
AD: Aggregated data
CP: Chronic pain
IMMPACT: Initiative on Methods, Measurement, and Pain Assessment in Clinical Trials
IPD: Individual participant data
MA: Meta-analysis
PRISMA: Preferred Reporting Items for Systematic reviews and Meta-Analyses statement

## References

[1] Nicholas Michael, Vlaeyen Johan W.S., Rief Winfried, Barke Antonia, Aziz Qasim, Benoliel Rafael, Cohen Milton, Evers Stefan, Giamberardino Maria Adele, Goebel Andreas, Korwisi Beatrice, Perrot Serge, Svensson Peter, Wang Shuu-Jiun, Treede Rolf-Detlef (2019). The IASP classification of chronic pain for ICD-11. PAIN.

[2] Leadley R. M., Armstrong N., Lee Y. C., Allen A., Kleijnen J. (2012). Chronic Diseases in the European Union: The Prevalence and Health Cost Implications of Chronic Pain. Journal of Pain & Palliative Care Pharmacotherapy.

[3] Gureje O, Von Korff M, Simon GE, Gater R. Persistent pain and well-being. JAMA. 1998;280(2):147 Available from: https://jamanetwork.com/journals/jama/fullarticle/187723 TS - CrossRef. 10.1001/jama.280.2.147 9669787

[4] IASP. Unrelieved pain is a major global healthcare problem. Vol. 15, Iasp. 2004. Available from: https://www.iasp-pain.org/files/Content/ContentFolders/GlobalYearAgainstPain2/20042005RighttoPainRelief/factsheet.pdf

[5] Breivik H, Eisenberg E, O’Brien T (2013). The individual and societal burden of chronic pain in Europe: the case for strategic prioritisation and action to improve knowledge and availability of appropriate care. BMC Public Health.

[6] Rice ASC, Smith BH, Blyth FM. Pain and the global burden of disease. Pain. 2016;157(4):791–6 Available from: http://content.wkhealth.com/linkback/openurl?sid=WKPTLP:landingpage&an=00006396-201604000-00006. [cited 2017 Sep 18]. 10.1097/j.pain.0000000000000454 26670465

[7] James SL, Abate D, Abate KH, Abay SM, Abbafati C, Abbasi N (2018). Global, regional, and national incidence, prevalence, and years lived with disability for 354 diseases and injuries for 195 countries and territories, 1990–2017: a systematic analysis for the Global Burden Of Disease Study 2017. Lancet..

[8] Gatchel Robert J., Peng Yuan Bo, Peters Madelon L., Fuchs Perry N., Turk Dennis C. (2007). The biopsychosocial approach to chronic pain: Scientific advances and future directions. Psychological Bulletin.

[9] Turk DC, Wilson HD, Cahana A (2011). Treatment of chronic non-cancer pain. Lancet.

[10] Hayes SC, Strosahl K, Wilson KG (1999). Acceptance and commitment therapy: an experiential approach to behavior change.

[11] Hayes Steven C. (2004). Acceptance and commitment therapy, relational frame theory, and the third wave of behavioral and cognitive therapies. Behavior Therapy.

[12] Dahl J, Wilson KG, Nilsson A. Acceptance and commitment therapy and the treatment of persons at risk for long-term disability resulting from stress and pain symptoms: a preliminary randomized trial. Behav Ther. 2004 ;35(4):785–801. Available from: https://www.sciencedirect.com/science/article/pii/S0005789404800200/pdf?md5=07ce4249a4fab54dc41d138ad24d6287&pid=1-s2.0-S0005789404800200-main.pdf. [cited 2017 Nov 1]

[13] Wicksell R. K., Kemani M., Jensen K., Kosek E., Kadetoff D., Sorjonen K., Ingvar M., Olsson G. L. (2012). Acceptance and commitment therapy for fibromyalgia: A randomized controlled trial. European Journal of Pain.

[14] Kemani Mike K., Olsson Gunnar L., Lekander Mats, Hesser Hugo, Andersson Erik, Wicksell Rikard K. (2015). Efficacy and Cost-effectiveness of Acceptance and Commitment Therapy and Applied Relaxation for Longstanding Pain. The Clinical Journal of Pain.

[15] Luciano Juan V., Guallar José A., Aguado Jaume, López-del-Hoyo Yolanda, Olivan Bárbara, Magallón Rosa, Alda Marta, Serrano-Blanco Antoni, Gili Margalida, Garcia-Campayo Javier (2014). Effectiveness of group acceptance and commitment therapy for fibromyalgia: A 6-month randomized controlled trial (EFFIGACT study). Pain.

[16] McCracken Lance M., Sato Ayana, Taylor Gordon J. (2013). A Trial of a Brief Group-Based Form of Acceptance and Commitment Therapy (ACT) for Chronic Pain in General Practice: Pilot Outcome and Process Results. The Journal of Pain.

[17] Wetherell Julie Loebach, Afari Niloofar, Rutledge Thomas, Sorrell John T., Stoddard Jill A., Petkus Andrew J., Solomon Brittany C., Lehman David H., Liu Lin, Lang Ariel J., Atkinson Hampton J. (2011). A randomized, controlled trial of acceptance and commitment therapy and cognitive-behavioral therapy for chronic pain. Pain.

[18] Plumb Vilardaga JC. Acceptance and commitment therapy for longstanding chronic pain in a community-based outpatient group setting. Diss Abstr Int Sect B Sci Eng. 2013;74(5–B(E)):No-Specified. Available from: http://gateway.proquest.com/openurl?url_ver=Z39.88-2004&rft_val_fmt=info:ofi/fmt:kev:mtx:dissertation&res_dat=xri:pqm&rft_dat=xri:pqdiss:3550275%5Cn; http://ovidsp.ovid.com/ovidweb.cgi?T=JS&PAGE=reference&D=psyc10&NEWS=N&AN=2013-99220-469.

[19] Mo'tamedi Hadi, Rezaiemaram Payman, Tavallaie Abaas (2012). The Effectiveness of a Group-Based Acceptance and Commitment Additive Therapy on Rehabilitation of Female Outpatients With Chronic Headache: Preliminary Findings Reducing 3 Dimensions of Headache Impact. Headache: The Journal of Head and Face Pain.

[20] Herbert Matthew Scott, Afari Niloofar, Liu Lin, Heppner Pia, Rutledge Thomas, Williams Kathryn, Eraly Satish, VanBuskirk Katie, Nguyen Cathy, Bondi Mark, Atkinson J. Hampton, Golshan Shahrokh, Wetherell Julie Loebach (2017). Telehealth Versus In-Person Acceptance and Commitment Therapy for Chronic Pain: A Randomized Noninferiority Trial. The Journal of Pain.

[21] Alonso-Fernández M, López-López A, Losada A, González JL, Wetherell JL. Acceptance and commitment therapy and selective optimization with compensation for institutionalized older people with chronic pain. Pain Med. 2015;17(2):n/a. Available from: https://academic.oup.com/painmedicine/article-lookup/doi/10.1111/pme.12885. [cited 2017 Nov 2] 10.1111/pme.12885 26304771

[22] Alonso-Fernandez M, Lopez-Lopez A, Losada A, Gonzalez JL, Wetherell JL. Acceptance and commitment therapy and selective optimization with compensation for institutionalized older people with chronic pain. Behav Psychol / Psicol Conduct Rev Int Clin y la Salud. 2013; Available from: http://onlinelibrary.wiley.com/journal/10.1111/(ISSN)1526-4637%5Cn ;http://eproxy.lib.hku.hk/login?url=http://ovidsp.ovid.com/ovidweb.cgi?T=JS&CSC=Y&NEWS=N&PAGE=fulltext&D=emed18&AN=605802722%5Cn; http://metadata.lib.hku.hk:3410/hku?sid=OVID:embase&id=pmid:&i. 10.1111/pme.12885 26304771

[23] Pincus T, Anwar S, McCracken LM, McGregor A, Graham L, Collinson M (2015). Delivering an optimised behavioural intervention (OBI) to people with low back pain with high psychological risk; results and lessons learnt from a feasibility randomised controlled trial of contextual cognitive behavioural therapy (CCBT) vs. physiotherap. BMC Musculoskelet Disord.

[24] Thorsell Jenny, Finnes Anna, Dahl JoAnne, Lundgren Tobias, Gybrant Maria, Gordh Torsten, Buhrman Monica (2011). A Comparative Study of 2 Manual-based Self-Help Interventions, Acceptance and Commitment Therapy and Applied Relaxation, for Persons With Chronic Pain. The Clinical Journal of Pain.

[25] Johnston Marnie, Foster Mary, Shennan Jeannette, Starkey Nicola J., Johnson Anders (2010). The Effectiveness of an Acceptance and Commitment Therapy Self-help Intervention for Chronic Pain. The Clinical Journal of Pain.

[26] Yang S-Y, Moss-Morris R, LM MC (2017). iACT-CEL: a feasibility trial of a face-to-face and internet-based acceptance and commitment therapy intervention for chronic pain in Singapore. Pain Res Treat.

[27] Scott W., Chilcot J., Guildford B., Daly-Eichenhardt A., McCracken L.M. (2018). Feasibility randomized-controlled trial of online Acceptance and Commitment Therapy for patients with complex chronic pain in the United Kingdom. European Journal of Pain.

[28] Lin J, Paganini S, Sander L, Lüking M, Ebert DD, Buhrman M (2017). An internet-based intervention for chronic pain: a three-arm randomized controlled study of the effectiveness of guided and unguided acceptance and commitment therapy. Dtsch Arztebl Int.

[29] Buhrman Monica, Skoglund Astrid, Husell Josefin, Bergström Kristina, Gordh Torsten, Hursti Timo, Bendelin Nina, Furmark Tomas, Andersson Gerhard (2013). Guided internet-delivered acceptance and commitment therapy for chronic pain patients: A randomized controlled trial. Behaviour Research and Therapy.

[30] Trompetter HR, Bohlmeijer ET, Veehof MM (2015). Schreurs KMG. Internet-based guided self-help intervention for chronic pain based on acceptance and commitment therapy: a randomized controlled trial. J Behav Med.

[31] The British Pain Society (2013). Guidelines for pain management programmes for adults an evidence-based review prepared on behalf of the British Pain Society.

[32] Feliu Soler A, Montesinos F, Gutiérrez-Martínez O, Scott W, McCracken L, Luciano J (2018). Current status of acceptance and commitment therapy for chronic pain: a narrative review. J Pain Res.

[33] Veehof M. M., Trompetter H. R., Bohlmeijer E. T., Schreurs K. M. G. (2016). Acceptance- and mindfulness-based interventions for the treatment of chronic pain: a meta-analytic review. Cognitive Behaviour Therapy.

[34] Sturgeon JA. Psychological therapies for the management of chronic pain. Psychol Res Behav Manag. 2014;7:115–24 Available from: https://www.ncbi.nlm.nih.gov/pmc/articles/PMC3986332/. [cited 2017 Aug 30]. 10.2147/PRBM.S44762 PMC3986332 24748826

[35] Hughes Laura S., Clark Jodi, Colclough Janette A., Dale Elizabeth, McMillan Dean (2017). Acceptance and Commitment Therapy (ACT) for Chronic Pain. The Clinical Journal of Pain.

[36] Yang S-Y, McCracken LM. Acceptance and commitment therapy for chronic pain. Journal of Clinical Outcomes Management 21.3 (2014): 134-144. Available from https://www.mdedge.com/jcomjournal/article/147127/pain/acceptance-and-commitment-therapy-chronic-pain.

[37] Turk DC (2005). The potential of treatment matching for subgroups of patients with chronic pain: lumping versus splitting. Clin J Pain.

[38] Vlaeyen Johan W. S., Morley Stephen (2005). Cognitive-Behavioral Treatments for Chronic Pain. The Clinical Journal of Pain.

[39] Gilpin HR, Keyes A, Stahl DR, Greig R, LM MC (2017). Predictors of treatment outcome in contextual cognitive and behavioral therapies for chronic pain: a systematic review. J Pain.

[40] Gurung Tara, Ellard David R., Mistry Dipesh, Patel Shilpa, Underwood Martin (2015). Identifying potential moderators for response to treatment in low back pain: A systematic review. Physiotherapy.

[41] Miles CL, Pincus T, Carnes D, Homer KE, Taylor SJC, Bremner SA, et al. Can we identify how programmes aimed at promoting self-management in musculoskeletal pain work and who benefits? A systematic review of sub-group analysis within RCTs. Eur J Pain. 2011;15(8):775.e1–775.e11. Available from: http://doi.wiley.com/10.1016/j.ejpain.2011.01.016. [cited 2017 Oct 26] 10.1016/j.ejpain.2011.01.016 21354838

[42] McCracken Lance M., Turk Dennis C. (2002). Behavioral and Cognitive–Behavioral Treatment for Chronic Pain. Spine.

[43] Wertli MM, Rasmussen-Barr E, Held U, Weiser S, Bachmann LM, Brunner F (2014). Fear-avoidance beliefs—a moderator of treatment efficacy in patients with low back pain: a systematic review. Spine J.

[44] Hurley MV, Walsh NE, Mitchell HL, Pimm TJ, Patel A, Williamson E (2007). Clinical effectiveness of a rehabilitation program integrating exercise, self-management, and active coping strategies for chronic knee pain: a cluster randomized trial. Arthritis Rheum.

[45] Riley R. D., Lambert P. C., Abo-Zaid G. (2010). Meta-analysis of individual participant data: rationale, conduct, and reporting. BMJ.

[46] Clarke MJ, Stewart LA (1994). Systematic reviews: obtaining data from randomised controlled trials: How much do we need for reliable and informative meta-analyses?. BMJ.

[47] Wicksell Rikard K., Olsson Gunnar L., Hayes Steven C. (2010). Psychological flexibility as a mediator of improvement in Acceptance and Commitment Therapy for patients with chronic pain following whiplash. European Journal of Pain.

[48] Hayes SC, Villatte M, Levin M, Hildebrandt M (2011). Open, aware, and active: contextual approaches as an emerging trend in the behavioral and cognitive therapies. Annu Rev Clin Psychol.

[49] Hayes Steven C., Luoma Jason B., Bond Frank W., Masuda Akihiko, Lillis Jason (2006). Acceptance and Commitment Therapy: Model, processes and outcomes. Behaviour Research and Therapy.

[50] Turk DC, Dworkin RH, Allen RR, Bellamy N, Brandenburg N, Carr DB, et al. Core outcome domains for chronic pain clinical trials: IMMPACT recommendations. Pain. 2003;106(3):337–45 Available from: https://journals.lww.com/pain/Abstract/2003/12000/Core_outcome_domains_for_chronic_pain_clinical.14.aspx TS - CrossRef. 10.1016/j.pain.2003.08.001 14659516

[51] Dworkin RH, Turk DC, Farrar JT, Haythornthwaite JA, Jensen MP, Katz NP, et al. Core outcome measures for chronic pain clinical trials: IMMPACT recommendations. Pain. 2005;113(1–2):9–19 Available from: https://journals.lww.com/pain/Citation/2005/01000/Core_outcome_measures_for_chronic_pain_clinical.5.aspx. [cited 2017 Sep 20]. 10.1016/j.pain.2004.09.012 15621359

[52] Liberati A, Altman DG, Tetzlaff J, Mulrow C, Gøtzsche PC, Ioannidis JPA, et al. The PRISMA statement for reporting systematic reviews and meta-analyses of studies that evaluate health care interventions: explanation and elaboration. Vol. 6, PLoS Med;2009 . p. e1000100. Available from: http://dx.plos.org/10.1371/journal.pmed.1000100. [cited 2017 Aug 2]. John Wiley & Sons 10.1371/journal.pmed.1000100 PMC2707010 19621070

[53] Stewart LA, Clarke M, Rovers M, Riley RD, Simmonds M, Stewart G (2015). Preferred reporting items for a systematic review and meta-analysis of individual participant data: The PRISMA-IPD statement. JAMA.

[54] Dissemination C for R and (2009). CRD’s guidance for undertaking reviews in health care.

[55] Dworkin RH, Turk DC, Wyrwich KW, Beaton D, Cleeland CS, Farrar JT (2008). Interpreting the clinical importance of treatment outcomes in chronic pain clinical trials: IMMPACT recommendations. J Pain.

[56] Wicksell RK, Olsson GL, Melin L (2009). The chronic pain acceptance questionnaire (CPAQ)-further validation including a confirmatory factor analysis and a comparison with the Tampa Scale of Kinesiophobia. Eur J Pain.

[57] McCracken Lance M., DaSilva Philomena, Skillicorn Beth, Doherty Richard (2014). The Cognitive Fusion Questionnaire. The Clinical Journal of Pain.

[58] Yu Lin, Norton Sam, McCracken Lance M. (2017). Change in “Self-as-Context” (“Perspective-Taking”) Occurs in Acceptance and Commitment Therapy for People With Chronic Pain and Is Associated With Improved Functioning. The Journal of Pain.

[59] Yu L, Norton S, Harrison A, McCracken LM (2015). In search of the person in pain: a systematic review of conceptualization, assessment methods, and evidence for self and identity in chronic pain. J Context Behav Sci.

[60] LM MC, Chilcot J, Norton S (2015). Further development in the assessment of psychological flexibility: a shortened Committed Action Questionnaire (CAQ-8). Eur J Pain (United Kingdom).

[61] McCracken Lance M. (2013). Committed Action: An Application of the Psychological Flexibility Model to Activity Patterns in Chronic Pain. The Journal of Pain.

[62] Scott Whitney, McCracken Lance M., Norton Sam (2015). A Confirmatory Factor Analysis of Facets of Psychological Flexibility in a Sample of People Seeking Treatment for Chronic Pain. Annals of Behavioral Medicine.

[63] Kerns Robert D., Turk Dennis C., Rudy Thomas E. (1985). The West Haven-Yale Multidimensional Pain Inventory (WHYMPI). Pain.

[64] Cleeland CS, Ryan KM. Pain assessment: global use of the Brief Pain Inventory. Ann Acad Med Singapore. 1994 ;23(2):129–138. Available from: https://psycnet.apa.org/record/2015-49222-001. [cited 2017 Oct 31] 8080219

[65] Chibnall John T., Tait Raymond C. (1994). The Pain Disability Index: Factor structure and normative data. Archives of Physical Medicine and Rehabilitation.

[66] Löwe B, Kroenke K, Herzog W, Gräfe K (2004). Measuring depression outcome with a brief self-report instrument: sensitivity to change of the Patient Health Questionnaire (PHQ-9). J Affect Disord.

[67] Zigmond A. S., Snaith R. P. (1983). The Hospital Anxiety and Depression Scale. Acta Psychiatrica Scandinavica.

[68] Löwe Bernd, Decker Oliver, Müller Stefanie, Brähler Elmar, Schellberg Dieter, Herzog Wolfgang, Herzberg Philipp Yorck (2008). Validation and Standardization of the Generalized Anxiety Disorder Screener (GAD-7) in the General Population. Medical Care.

[69] Luo Xuemei, Lynn George Mandy, Kakouras Ikey, Edwards Christopher L., Pietrobon Ricardo, Richardson William, Hey Lloyd (2003). Reliability, Validity, and Responsiveness of the Short Form 12-Item Survey (SF-12) in Patients With Back Pain. Spine.

[70] Richardson J, Iezzi A, Khan MA, Maxwell A (2014). Validity and reliability of the assessment of quality of life (AQoL)-8D multi-attribute utility instrument. Patient - Patient-Centered Outcomes Res.

[71] Rabin R, de Charro F (2001). EQ-SD: a measure of health status from the EuroQol group. Ann Med.

[72] Guy W (1976). ECDEU assessment manual for psychopharmacology. US Dept Health, Education, and Welfare publication (ADM).

[73] Bond FW, Hayes SC, Baer RA, Carpenter KM, Guenole N, Orcutt HK (2011). Preliminary psychometric properties of the Acceptance and Action Questionnaire-II: A revised measure of psychological inflexibility and experiential avoidance. Behav Ther.

[74] Vowles Kevin E., McCracken Lance M., McLeod Charlotte, Eccleston Christopher (2008). The Chronic Pain Acceptance Questionnaire: Confirmatory factor analysis and identification of patient subgroups. Pain.

[75] Gale EN, Funch DP (1984). Factors associated with successful outcome from behavioral therapy for chronic temporomandibular joint (TMJ) pain. J Psychosom Res.

[76] Dworkin Robert H., Richlin David M., Handlin David S., Brand Leonard (1986). Predicting treatment response in depressed and non-depressed chronic pain patients. Pain.

[77] De Rooij A, Van Der Leeden M, Roorda LD, MPM S, Dekker J (2013). Predictors of outcome of multidisciplinary treatment in chronic widespread pain: an observational study. BMC Musculoskelet Disord.

[78] Witt Claudia M., Schützler Lena, Lüdtke Rainer, Wegscheider Karl, Willich Stefan N. (2011). Patient Characteristics and Variation in Treatment Outcomes. The Clinical Journal of Pain.

[79] Turner Judith A., Holtzman Susan, Mancl Lloyd (2007). Mediators, moderators, and predictors of therapeutic change in cognitive–behavioral therapy for chronic pain. Pain.

[80] Svanberg M, Stålnacke B-M, Enthoven P, Brodda-Jansen G, Gerdle B, Boersma K. Impact of emotional distress and pain-related fear on patients with chronic pain: subgroup analysis of patients referred to multimodal rehabilitation. J Rehabil Med J Rehab Med. 2017;49(49):354–361. Available from: http://www.diva-portal.org/smash/get/diva2:1091396/FULLTEXT01.pdf%0A; https://www.medicaljournals.se/jrm/content/abstract/0.2340/16501977-2212. [cited 2017 Aug 30] 10.2340/16501977-2212 28352937

[81] Härkäpää K, Järvikoski A, Estlander A-M (1996). Health optimism and control beliefs as predictors for treatment outcome of a multimodal back treatment program. Psychol Heal.

[82] Ahmed I., Sutton A. J., Riley R. D. (2012). Assessment of publication bias, selection bias, and unavailable data in meta-analyses using individual participant data: a database survey. BMJ.

[83] Taylor RS, Piepoli MF, Smart N, Coats AJS, Ellis S, Dalal H (2014). Exercise training for chronic heart failure (ExTraMATCH II): protocol for an individual participant data meta-analysis. Int J Cardiol.

[84] Buffart LM, Kalter J, Chinapaw MJ, Heymans MW, Aaronson NK, Courneya KS (2013). Predicting OptimaL cAncer RehabIlitation and Supportive care (POLARIS): rationale and design for meta-analyses of individual patient data of randomized controlled trials that evaluate the effect of physical activity and psychosocial interventions on healt. Syst Rev.

[85] Higgins JPT, Altman DG, Gøtzsche PC, Jüni P, Moher D, Oxman AD, et al. The Cochrane Collaboration’s tool for assessing risk of bias in randomised trials. BMJ. 2011 ;343(oct18 2):d5928. Available from: https://www.bmj.com/content/343/bmj.d5928.long. [cited 2017 Oct 31] 10.1136/bmj.d5928 PMC3196245 22008217

[86] Guyatt GH, Oxman AD, Vist GE, Kunz R, Falck-Ytter Y, Alonso-Coello P, et al. GRADE: An emerging consensus on rating quality of evidence and strength of recommendations. Chinese J Evidence-Based Med. 2009 ;9(1):8–11. Available from: https://www.bmj.com/content/336/7650/924.long. [cited 2017 Oct 31]

[87] Guyatt Gordon H., Oxman Andrew D., Vist Gunn, Kunz Regina, Brozek Jan, Alonso-Coello Pablo, Montori Victor, Akl Elie A., Djulbegovic Ben, Falck-Ytter Yngve (2011). GRADE guidelines: 4. Rating the quality of evidence—study limitations (risk of bias). Journal of Clinical Epidemiology.

[88] Guyatt Gordon, Oxman Andrew D., Akl Elie A., Kunz Regina, Vist Gunn, Brozek Jan, Norris Susan, Falck-Ytter Yngve, Glasziou Paul, deBeer Hans (2011). GRADE guidelines: 1. Introduction—GRADE evidence profiles and summary of findings tables. Journal of Clinical Epidemiology.

[89] Stewart GB, Altman DG, Askie LM, Duley L, Simmonds MC, Stewart LA. Statistical analysis of individual participant data meta-analyses: a comparison of methods and recommendations for practice. Biondi-Zoccai G, editor. PLoS One. 2012 ;7(10):e46042. Available from: http://dx.plos.org/10.1371/journal.pone.0046042. [cited 2017 Aug 9] 10.1371/journal.pone.0046042 PMC3463584 23056232

[90] Simmonds MC, Higgins JPT, Stewart LA, Tierney JF, Clarke MJ, Thompson SG (2005). Meta-analysis of individual patient data from randomized trials: a review of methods used in practice. Clin Trials.

[91] R Core Team (2017). R: A language and environment for statistical computing.

[92] Cohen J (1988). Statistical power analysis for the behavioral sciences.

[93] O’Connor D, Green S, Higgins JP. Defining the review question and developing criteria for including studies. In: Cochrane Handbook for Systematic Reviews of Interventions: Cochrane Book Series. Wiley-Blackwell; 2008. p. 81–94.

[94] Stuck A. E, Rubenstein L. Z, Wieland D., Vandenbroucke J. P, Irwig L., Macaskill P., Berry G., Glasziou P., Seagroatt V., Stratton I., Egger M., Smith G. D., Minder C., Langhorne P., Song F., Gilbody S. (1998). Bias in meta-analysis detected by a simple, graphical. BMJ.

[95] Duval S, Tweedie R (2000). Trim and fill: a simple funnel-plot-based method of testing and adjusting for publication bias in meta-analysis. Biometrics.

[96] Burns JW. Mechanisms, mechanisms, mechanisms: it really does all boil down to mechanisms. Pain. 2016;157(11):2393–4 Available from: http://content.wkhealth.com/linkback/openurl?sid=WKPTLP:landingpage&an=00006396-201611000-00001. [cited 2017 Sep 18]. 10.1097/j.pain.0000000000000696 PMC5846486 27548048

[97] Lin Jiaxi, Klatt Laura-Isabelle, McCracken Lance M., Baumeister Harald (2018). Psychological flexibility mediates the effect of an online-based acceptance and commitment therapy for chronic pain. PAIN.

[98] Probst T, Baumeister H, mcCracken L, Lin J (2018). Baseline psychological inflexibility moderates the outcome pain interference in a randomized controlled trial on internet-based acceptance and commitment therapy for chronic pain. J Clin Med.

[99] Scott W, Hann KEJ, McCracken LM (2016). A comprehensive examination of changes in psychological flexibility following acceptance and commitment therapy for chronic pain. J Contemp Psychother.

[100] Vowles KE, Witkiewitz K, Sowden G, Ashworth J (2014). Acceptance and commitment therapy for chronic pain: evidence of mediation and clinically significant change following an abbreviated interdisciplinary program of rehabilitation. J Pain.

[101] Cederberg Jenny Thorsell, Cernvall Martin, Dahl JoAnne, von Essen Louise, Ljungman Gustaf (2015). Acceptance as a Mediator for Change in Acceptance and Commitment Therapy for Persons with Chronic Pain?. International Journal of Behavioral Medicine.

[102] Vowles KE, Sowden G, Ashworth J (2014). A comprehensive examination of the model underlying acceptance and commitment therapy for chronic pain. Behav Ther.

[103] Trompetter HR, Bohlmeijer ET, Fox J-PP, KMG S. Psychological flexibility and catastrophizing as associated change mechanisms during online acceptance & commitment therapy for chronic pain. Behav Res Ther. 2015;74:50–9 Available from: https://www.sciencedirect.com/science/article/pii/S000579671530036X?via%3Dihub. [cited 2018 Feb 1]. 10.1016/j.brat.2015.09.001 26409158

[104] Baranoff JA, Hanrahan SJ, Burke ALJ, Connor JP (2016). Changes in acceptance in a low-intensity, group-based acceptance and commitment therapy (ACT) chronic pain intervention. Int J Behav Med.

[105] Wicksell RK, Olsson GL, Hayes SC. Mediators of change in acceptance and commitment therapy for pediatric chronic pain. Pain. 2011;152(12):2792–801 Available from: https://journals.lww.com/pain/Abstract/2011/12000/Mediators_of_change_in_Acceptance_and_Commitment.19.aspx TS - CrossRef. 10.1016/j.pain.2011.09.003 21995881

