# Supplementary material for: Acceptance and commitment therapy for chronic pain: protocol of a systematic review and individual participant data meta-analysis
Source: Syst Rev. 2019 Jun 14;8:140. doi: 10.1186/s13643-019-1044-2 (PMC6570828; doi:10.1186/s13643-019-1044-2)
Supplement: Supplementary file 2 — ACT-CP-MA bibliographic database searches. (DOCX 20 kb) [file 13643_2019_1044_MOESM2_ESM.docx]

**Bibliographic database searches**

| **Ovid PsycINFO, MEDLINE(R) and Embase** | |
| --- | --- |
| 1 | (chronic* adj5 pain*).mp. |
| 2 | Eye Pain/ or Neck Pain/ or Pain/ or Nociceptive Pain/ or Facial Pain/ or Shoulder Pain/ or Myofascial Pain Syndromes/ or Pelvic Pain/ or Patellofemoral Pain Syndrome/ or Pelvic Girdle Pain/ or Abdominal Pain/ or Chronic Pain/ or Flank Pain/ or Low Back Pain/ or Musculoskeletal Pain/ or Chest Pain/ or Complex Regional Pain Syndromes/ or Visceral Pain/ or Back Pain/ |
| 3 | ((chronic* or back or musculoskel* or intractabl* or neuropath* or phantom limb or fantom limb or neck or myofasc* or temp? romandib* joint or central or post*stroke or complex or regional or spinal cord or idiopathic or shoulder or persistent) adj4 pain*).ab,ti. |
| 4 | (sciatica or back-ache or back*ache or lumbago or fibromyalg* or (trigemin* adj2 neuralg*) or (herp* adj2 neuralg*) or (diabet* adj2 neuropath*) or (reflex adj4 dystroph*) or (sudeck* adj2 atroph*) or causalg* or whip-lash or whip*lash or polymyalg* or (failed back adj4 surg*) or (failed back adj4 syndrome*) or arthritis or cumulative trauma or irritable bowel syndrome* or pancreatitis or peripheral vascular disease or repetitive strain injury or rheumatic or angina or interstitial cystitis or vestibulodynia).ab,ti. |
| 5 | 1 or 2 or 3 or 4 |
| 6 | randomized controlled trial.mp. [mp=title, abstract, heading word, drug trade name, original title, device manufacturer, drug manufacturer, device trade name, keyword, floating subheading word, candidate term word] |
| 7 | controlled clinical trial.mp. [mp=title, abstract, heading word, drug trade name, original title, device manufacturer, drug manufacturer, device trade name, keyword, floating subheading word, candidate term word] |
| 8 | randomized.mp. [mp=title, abstract, heading word, drug trade name, original title, device manufacturer, drug manufacturer, device trade name, keyword, floating subheading word, candidate term word] |
| 9 | placebo.mp. [mp=title, abstract, heading word, drug trade name, original title, device manufacturer, drug manufacturer, device trade name, keyword, floating subheading word, candidate term word] |
| 10 | randomly.mp. [mp=title, abstract, heading word, drug trade name, original title, device manufacturer, drug manufacturer, device trade name, keyword, floating subheading word, candidate term word] |
| 11 | trial.mp. [mp=title, abstract, heading word, drug trade name, original title, device manufacturer, drug manufacturer, device trade name, keyword, floating subheading word, candidate term word] |
| 12 | groups.mp. [mp=title, abstract, heading word, drug trade name, original title, device manufacturer, drug manufacturer, device trade name, keyword, floating subheading word, candidate term word] |
| 13 | 6 or 7 or 8 or 9 or 10 or 11 or 12 |
| 14 | (contextual cognitive behav* therap* or contextual behav* science).ti,ab. |
| 15 | ((acceptance adj5 therap*) or (acceptance adj5 strateg*) or (acceptance adj5 treatment*) or (acceptance adj5 intervention*) or (acceptance adj5 technique*)).ti,ab. |
| 16 | "acceptance and commitment therapy".mp. |
| 17 | (ACT adj5 therap*).ti,ab. |
| 18 | ((commitment adj5 therap*) or (commitment adj5 strateg*) or (commitment adj5 treatment*) or (commitment adj5 intervention*) or (commitment adj5 technique*)).ti,ab. |
| 19 | 15 or 16 or 17 or 18 or 14 |
| 20 | 5 and 13 and 19 |
| 21 | limit 20 to human |
| **Cochrane Library (EBM Reviews - Cochrane Central Register of Controlled Trials)** | |
| 1 | (chronic* near/5 pain*):ti in Trials |
| 2 | ("Eye Pain" or "Neck Pain" or "Pain" or "Nociceptive Pain" or "Facial Pain" or "Shoulder Pain" or "Myofascial Pain Syndromes" or "Pelvic Pain" or "Patellofemoral Pain Syndrome" or "Pelvic Girdle Pain" or "Abdominal Pain" or "Flank Pain" or "Low Back Pain" or "Musculoskeletal Pain" or "Chest Pain" or "Complex Regional Pain Syndromes" or "Visceral Pain" or "Back Pain"):ti,ab,kw in Trials |
| 3 | (chronic* or back or musculoskel* or intractabl* or neuropath* or phantom limb or fantom limb or neck or myofasc* or temp? romandib* joint or central or post*stroke or complex or regional or spinal cord or idiopathic or shoulder or persistent adj4 pain*):ti in Trials |
| 4 | sciatica or back-ache or back*ache or lumbago or fibromyalg* or (trigemin* near/2 neuralg*) or (herp* near/2 neuralg*) or (diabet* near/2 neuropath*) or (reflex near/4 dystroph*) or (sudeck* near/2 atroph*) or causalg* or whip-lash or whip*lash or polymyalg* or (failedback near/4 surg*) or (failedback near/4 syndrome*) or arthritis or cumulative trauma or irritable bowel syndrome* or pancreatitis or peripheral vascular disease or repetitive strain injury or rheumatic or angina or interstitial cystitis or vestibulodynia:ti,ab,kw in Trials |
| 5 | #1 or #2 0r #3 or #4 in Trials |
| 6 | (randomized controlled trial):ti,ab,kw in Trials |
| 7 | (controlled clinical trial):ti,ab,kw in Trials |
| 8 | randomized in Trials |
| 9 | placebo in Trials |
| 10 | randomly in Trials |
| 11 | trial in Trials |
| 12 | groups in Trials |
| 13 | #6 OR #7 OR #8 OR #9 #10 OR #11 OR #12 in Trials |
| 14 | contextual cognitive behav* therap* or contextual behav* science:ti,ab,kw in Trials |
| 15 | "acceptance and commitment therapy" in Trials |
| 16 | (acceptance NEAR/5therap*) or (acceptance near/5 strateg*) or (acceptance NEAR/5treatment*) or (acceptance near/5 intervention*) or (acceptance near/5 technique*):ti,ab,kw in Trials |
| 17 | ACT near/5 therap*:ti,ab,kw in Trials |
| 18 | (commitment near/5 therap*) or (commitment near/5 strateg*) or (commitment near/5 treatment*) or (commitment near/5 intervention*) or (commitment near/5 technique*) .:ti,ab,kw in Trials |
| 19 | #14 or #15 or #16 or #17 or #18 in Trials |
| 20 | #5 and #13 and #19 |
| 21 | animals NOT humans (Word variations have been searched) |
| 22 | #20 NOT 22 in Trials |
