# Supplementary material for: Acceptance and commitment therapy for chronic pain: protocol of a systematic review and individual participant data meta-analysis
Source: Syst Rev. 2019 Jun 14;8:140. doi: 10.1186/s13643-019-1044-2 (PMC6570828; doi:10.1186/s13643-019-1044-2)
Supplement: Supplementary file 3 — ACT Pain IPD invitation letter. (DOCX 26 kb) [file 13643_2019_1044_MOESM3_ESM.docx]

**To: Corresponding author**

**CC:** [Jiaxi.lin@psychologie.uni-freiburg.de](mailto:Jiaxi.lin@psychologie.uni-freiburg.de)

**Subject:** Acceptance and Commitment Therapies for chronic pain: individual patient data meta-analysis

Dear **Corresponding author**,

I hope you are well. We, Jiaxi Lin and some other colleagues in my section and the Universities Ulm and Freiburg, Germany, are planning to conduct an individual patient data meta-analysis on Acceptance and Commitment therapy for chronic pain (ACT-CP-MA).

Since a number of important trials of ACT in chronic pain have been published within the last two decades, we would like to bring together all the trials within a merged dataset. Based on this dataset, we can conduct standardised statistical analyses on the effect and moderators and predictors of treatment effects. We are contacting all lead investigators of trials of ACT in chronic pain to invite them to participate in our ACT-CP-MA project.

Your trial (Reference) was identified in our review on existing RCTs on ACT for chronic pain. We would like to invite you to join our project as a collaborator and make available the individual patient dataset from your trial for the purpose of this project.

Attached, you can find a document with detailed information on methodological aspects, your involvement in the project, and how we will handle your data.

We very much look forward to hearing from you, and hope you will wish to be involved in this important international collaboration in the field of ACT for chronic pain.

Please contact Dr. Jiaxi Lin (principle investigator in this project, email address in CC) for further communication and questions.

Yours sincerely

Lance McCracken

And on behalf of the ACT-CP-MA Group

Dr Jiaxi Lin, King's College London, UK and from 2018: University of Freiburg, Germany

Dr Whitney Scott, King's College London, UK

Matthias Domhardt, University of Ulm, Germany

Dr. Lewis Carpenter, King's College London, UK

Dr Sam Norton, King's College London, UK

Professor Dr Harald Baumeister, University of Ulm, Germany

**Individual Participant Data Meta-analysis of Acceptance and Commitment therapy for chronic pain (ACT-CP-MA)**

**General information on the project**

**How does an individual participant data meta-analysis differ from a standard meta-analysis?**

Traditional meta-analysis methods involve aggregating trial level results, typically obtained from summary data reported in published papers. An alternative and increasingly popular approach is meta-analysis of individual participant data (IPD), in which the raw individual level data for each study are obtained and used for analysis.

IPD meta-analyses offer a number of advantages over traditional meta-analyses, including:

- statistical analysis can be standardized across studies – all data can be analyzed with the same method
- superior power to assess the treatment effects, including in relation to specific subgroups, based on participant characteristics, and on differences in treatment methods or modes of delivery, such as one-to-one, group, individual, or online
- missing data can be observed and accounted for at the individual level.

**What data am I being asked to share?**

The initial phase of ACT-CP-MA includes gaining access to individual participant data for the following variables from your trial:

- Socio-demographic variables (age, gender, employment status, level of education, relationship status)
- Pain-related factors (pain duration, baseline pain interference and intensity);
- Primary outcome (pain interference, for example, MPI or BPI);
- Secondary outcomes (pain intensity, depression and anxiety (emotional functioning), health-related quality of life, pain acceptance, psychological flexibility).

**Do I need ethics permission to make my data available?**

No. Participants have consented to participate in their original trial. Given that the analyses proposed by ACT-CP-MA are simply an extension of the core analysis of the constituent trials and that we only request anonymized data, we do not anticipate that additional ethical permission will be required. However, we cannot include the data of trials in ACT-CP-MA if ethics approval was not obtained for the original study.

**Will my data be securely held?**

Yes. We will ensure that datasets shared as part of the project include no participant-identifiable information (such as names and addresses), and that all data storage complies with the regulations governing research at University of Freiburg, Germany.

All data will be received and stored in a secure database at the University of Freiburg, Germany. Both the coordinating center at University of Freiburg, Germany, and King’s College London in the UK will hold a copy of the dataset.

**How should I organize the transfer of my data?**

We will work with you and each individual trial site to determine the best way to transfer your participant level data.

**What will be done with the data?**

Individual trial datasets will be combined into one overall dataset with standardized variables, working with individual trial authors to ensure that standardization of variables is conducted with fidelity and to check that our analyses of individual datasets are consistent with the published results from the trial. Once the combined dataset has been developed, the first phase of the ACT-CP-MA data analysis will be to address the following three primary objectives:

(1) To conduct an IPD-MA to evaluate the effects of ACT on pain interference and other available key outcomes of individuals with chronic pain

(2) To identify factors that appear to moderate treatment effects of ACT, including

1. Individual baseline characteristics or measures, such as socio-demographic, population, pain-related characteristics, baseline outcomes, or facets of psychological flexibility
2. Treatment characteristics, such as traditional one-to-one face-to-face ACT, group-delivered face-to-face ACT, or guided/unguided internet intervention, number of sessions, number of total hours, treatment providers, and the like

**Who owns the data?**

Data from individual datasets will remain the property of the ACT-CP-MA collaborators who have provided IPD. You remain the custodian for your own data and retain the right to withdraw your data from the ACT-CP-MA collaboration at any time.

**How will I be acknowledged on presentations and publications based on the ACT for chronic pain data?**

All publications from the combined data will include the ACT-CP-MA research team and all collaborators. Where collaborators involve multiple individual authors per trial, authorship will be restricted to a maximum of two authors per trial included. Requirements for authorship are those of the International Committee of Medical Journal Editors (http://www.icmje.org). Before publication of any ACT-CP-MA manuscripts, drafts will be circulated for comment, revision and approval. Publications using these data will be authored on behalf of the ACT-CP-MA collaboration, either with specific named authors, or on behalf of the ACT-CP-MA collaboration as a whole, with names of other participating collaborators listed in the acknowledgements.

**What is the scope of ACT-CP-MA?**

We regard ACT-CP-MA as more than a single IPD project. With the high potential of ACT in treating chronic pain, multiple RCTs will be published in the years to come and further research is needed in many aspects that are not yet covered in ACT-CP-MA. For example, with increasing data from future RCTs, treatment processes across multiple trials can be analyzed in mediation models. Therefore, ACT-CP-MA is the beginning of a long-term collaboration with a high potential to provide systematic reviews on the effectiveness and mediators, predictors and moderators of treatment effect with regular updates.

**Is there a possibility to actively collaborate?**

You are very welcome to actively collaborate with us in addition to the provision of your data. As there will be many work packages within the ACT-CP-MA project, it would be best to contact Dr. Jiaxi Lin (principle investigator in this project, [jiaxi.lin@psychologie.uni-freiburg.de](mailto:jiaxi.lin@psychologie.uni-freiburg.de)) to sort out how to contribute in ACT-CP-MA.
